# Pharmacokinetics, Tissue Distribution and Excretion of a Novel Diuretic (PU-48) in Rats

**DOI:** 10.3390/pharmaceutics10030124

**Published:** 2018-08-08

**Authors:** Zhi-Yuan Zhang, Hua Zhang, Dan Liu, Ying-Yuan Lu, Xin Wang, Pu Li, Ya-Qing Lou, Bao-Xue Yang, Ya-Xin Lou, Chuang Lu, Qiang Zhang, Guo-Liang Zhang

**Affiliations:** 1 Department of Pharmacology, School of Basic Medical Sciences, Peking University, No.38 Xue-Yuan Road, Beijing 100191, China; zzy_705@163.com (Z.-Y.Z.); Luyingyuan2005@126.com (Y.-Y.L.); wangxinpku@bjmu.edu.cn (X.W.); lipu9909@126.com (P.L.); cdcdname@21cn.com (Y.-Q.L.); baoxue@bjmu.edu.cn (B.-X.Y.); 2 Department of Pharmaceutics, School of Pharmaceutical Sciences, Peking University, Beijing 100191, China; zhangpharm@bjmu.edu.cn; 3 Proteomics Laboratory, Medical and Health Analysis Center, Peking University, Beijing 100191, China; liudan@bjmu.edu.cn (D.L.); louyx@bjmu.edu.cn (Y.-X.L.); 4 Department of Drug Metabolism & Pharmacokinetics (DMPK), Sanofi, Waltham, MA 02451, USA; chuanglu7@gmail.com

**Keywords:** methyl 3-amino-6-methoxythieno [2,3-b] quinolone-2-carboxylate (PU-48), plasma pharmacokinetics, tissue distribution, excretion, plasma protein binding, rat

## Abstract

Methyl 3-amino-6-methoxythieno [2,3-b] quinoline-2-carboxylate (PU-48) is a novel diuretic urea transporter inhibitor. The aim of this study is to investigate the profile of plasma pharmacokinetics, tissue distribution, and excretion by oral dosing of PU-48 in rats. Concentrations of PU-48 within biological samples are determined using a validated high performance liquid chromatography-tandem mass spectrometry (LC-MS/MS) method. After oral administration of PU-48 (3, 6, and 12 mg/kg, respectively) in self-nanomicroemulsifying drug delivery system (SNEDDS) formulation, the peak plasma concentrations (*C*_max_), and the area under the curve (AUC_0–∞_) were increased by the dose-dependent and linear manner, but the marked different of plasma half-life (*t*_1/2_) were not observed. This suggests that the pharmacokinetic profile of PU-48 prototype was first-order elimination kinetic characteristics within the oral three doses range in rat plasma. Moreover, the prototype of PU-48 was rapidly and extensively distributed into thirteen tissues, especially higher concentrations were detected in stomach, intestine, liver, kidney, and bladder. The total accumulative excretion of PU-48 in the urine, feces, and bile was less than 2%. This research is the first report on disposition via oral administration of PU-48 in rats, and it provides important information for further development of PU-48 as a diuretic drug candidate.

## 1. Introduction

**Figure 1:**
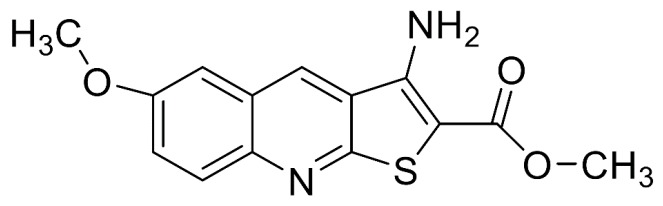
Chemical structure of methyl 3-amino-6-methoxythieno [2,3-b] quinoline-2-carboxylate (PU-48).

The methyl 3-amino-6-methoxythieno [2,3-b] quinoline-2-carboxylate (PU-48, Figure 1) is a novel thienoquinolin diuretic agent, and this compound is able to induce a diuresis by inhibiting urea transporters (UTs) in the inner medullary collecting duct (IMCD) [1,2,3]. Urea transporters are a family of membrane proteins that facilitate the passive transport of urea due to the countercurrent multiplication mechanism for urinary concentration in IMCD [4,5]. Recently, two main UT subfamilies including UT-A and UT-B have been identified in mammalians [6,7]. The UT-A isoforms is mainly expressed in renal IMCD cells, and the UT-B isoform is predominantly distributed in multiple extrarenal tissues such as vascular endothelium and erythrocytes [8,9,10,11,12,13].

It has been reported that PU-48 exhibits more potent diuretic activity and higher selective inhibition towards IMCD urea transporter UT-As in rodents, when compared to the other thienoquinolin lead compounds for non-selective inhibition of both UT-B and UT-A isoforms [14,15,16,17,18,19,20]. As this compound did not change serum levels of sodium, chloride, or potassium, PU-48 has fewer adverse effect on electrolyte disturbance than conventional diuretics such as dihydrochlorothiazide or furosemide [21,22,23,24]. Due to the unique pharmacological mechanism, induced by the salt-sparing diuretic action (urearetics), PU-48 has high potential to be a promising novel diuretic candidate.

As shown in our earlier research, a sensitive and selective high-performance liquid chromatographic-tandem mass spectrometry (LC-MS/MS) method for determination of PU-48 in plasma was developed and validated [25]. However, expanding pharmacokinetic research, has not consisted of work relating to the rate and the extent of absorption and elimination of PU-48. Therefore, the objective of this study is to evaluate the profiles of the disposition including plasma pharmacokinetics, tissue distribution, and excretion of PU-48 after oral administration with the SNEDDS formulation in rats.

## 2. Materials and Methods

### 2.1. Chemicals and Reagents

Methyl 3-amino-6-methoxythieno [2,3-b] quinoline-2-carboxylate (PU-48) (purity > 99%) was obtained from the Department of Chemical Biology and Pharmaceutics, School of Pharmaceutical Sciences, Peking University (Beijing, China). The self-nanomicroemulsion drug delivery systems (SNEDDS) formulation of PU-48 (1.2 mg/mL) was prepared by the Department of Pharmaceutics, School of Pharmaceutical Sciences, Peking University (Beijing, China). Megestrol acetate was used as the internal standard (IS) and was purchased from the National Institute for the Control of Pharmaceutical and Biological Products (Beijing, China). Formic acid was purchased from Sigma–Aldrich (St. Louis, MA, USA). Acetonitrile and methanol (high-performance liquid chromatographic (HPLC) grade) were purchased from Fisher Scientific (Fair Lawn, NJ, USA). Extraction agent ethyl acetate was purchased from Merck Schuchardt OHG (HPLC grade, Darmstadt, Germany). Deionized water was purified by a Millipore water purification system (Millipore, MA, USA).

### 2.2. Animal Handling

Male Sprague-Dawley rats (weighing 220 ± 20 g) were provided by the Department of Experimental Animal, Peking University (Beijing, China). Environmental controls for the animal room were set at 22 ± 3 °C, a 12 h light-dark cycle and rats were fed with free access to food and drinking water before the experiment. Animal experimental were approved by the Animal Ethics Committee of Peking University Health Science Center (the registration number: LA2016172, 2 February 2016).

### 2.3. LC-MS/MS Analysis and Method Validation 

The method for quantification PU-48 in rat samples (plasma, thirteen tissues, urine and bile) has been developed and validated in our preliminary experiment [25]. Briefly, PU-48 was quantitative analyzed by the liquid chromatography-electrospray ionization source-triple quadrupole tandem mass spectrometer (LC-MS/MS) system, which consisted of the Shimazdu LC-20AD pumpers (Shimadzu, Kyoto, Japan), a 20 μL loop and a SPD-M20A PDA detector. Chromatographic separation was accomplished on a Shimazdu Inertsil ODS-4 C18 column (2.1 × 100 mm, 3 µm, Shimadzu) and coupled to the API4000 mass spectrometer detector equipped with the Analyst 1.6 software (AB Sciex, Redwood City, CA, USA). The monitored ion transitions were mass-to-charge ratio (*m*/*z*) 289.1 → 229.2 for PU-48 and *m*/*z* 385.3 → 267.1 for the internal standard under positive mode. The mobile phase consisted of (A) water, and (B) acetonitrile, both containing 0.05% formic acid using the following gradient elution: 0–2 min (20% B), 2–8 min (20–100% B), 8–9.5 min (100% B), 9.5–13 min (20% B). Sample of 5 μL was injected. The quantification analysis of PU-48 in plasma, urine, bile and tissues (liver, muscle, and adipose tissue) were evaluated for specificity, linearity, precision, accuracy and recovery (See Supplementary Materials Tables S1–S3). 

### 2.4. HPLC Analysis and Method Validation

The concentration of PU-48 prototype in biological sample (feces and plasma protein binding analysis) was determined using a validated high-performance liquid chromatographic (HPLC) method our preliminary experiment (See Supplementary Materials HPLC Analysis and Method Validation). Briefly, the quantitative analysis of PU-48 was performed using a HPLC consistent of model 510 pump with a model 2487 ultraviolet detector, a model Rheodyne 7725 injector, and a column oven. The analytical conditions were as follows—The detection wavelength—293 nm; analytical column—a reversed-phase Acclaim C18 (4.6 mm × 250 mm, 5.0 µm, Dionex, Sunnyvale, CA, USA); column temperature—25 °C; mobile phase—acetonitrile—distilled water at a ratio of 60:40 (*v*/*v*); flow rate—1 mL/min. In this study, the methods for the feces samples were evaluated for specificity, linearity, precision, accuracy, and recovery (See Supplementary Materials Tables S4–S6).

### 2.5. Preparation of Plasma, Tissue, Urine, Feces and Bile Samples

Internal standard (IS, 25 μL) was added to 50 μL plasma, urine, or bile sample in a clean tube, placed into a water bath at 37 °C, and dried under a gentle stream of nitrogen gas, followed by the addition of 0.5 mL of ethyl acetate for liquid-liquid extraction. The mixture was shaken by a vortex mixer for 3 min, and then centrifuged at 8000 rpm (4200× *g*) for 10 min at 4 °C. After centrifugation, the upper organic phase was transferred into a clean centrifuge tube and then dried it again. The combined upper organic phases were evaporated to dryness at 37 °C under a gentle stream of nitrogen gas. The residue was reconstituted with 50 μL acetonitrile and then centrifuged at 13,000 rpm (12,000× *g*) for 10 min at 4 °C, the upper liquid was transferred into sample vials. Finally, 5 μL aliquots were injected into the LC–MS/MS system to analyze. Each tissue samples were homogenized in normal saline (0.9% NaCl, 1:3, *w*/*v*), and then centrifuged at 10,000× *g* for 10 min to get the supernatant. Other steps were the same as plasma sample preparation.

Feces samples were homogenized in 0.9% NaCl solution (1 g/10 mL), vortexed for 1 min and then centrifuged at 1200× *g* for 15 min to gather the supernatant. A volume of 25 μL of IS was added to 500 μL mixture in a clean tube, placed into a water bath at 37 °C and dried under a gentle stream of nitrogen gas, followed by the addition of 2 mL of ethyl acetate for twice liquid-liquid extraction (total 4 mL). The residue was reconstituted with 50 μL mobile phase and then 5 μL aliquots were injected into the HPLC system to analyze. 

### 2.6. Plasma Pharmacokinetics

After fasting overnight with free drinking water (12 h before the experiment), eighteen rats were divided randomly into three groups, and blood samples at each time point were collected from six rats. The rats were given three doses of PU-48 (3, 6, or 12 mg/kg, respectively, 1.2 mg/mL in the SNEDDS formulation) by oral gavage. Blood samples (0.3 mL) were collected from the ophthalmic venous plexus at the predose, 0.25, 0.5, 1, 2, 4, 6, 8, 12, 24, 36, and 48 h post-dosing (total 12 time points) in heparinized tubes. The blood samples were centrifuged at 2500 rpm (1200× *g*) for 15 min and the plasma was stored at −20 °C until analysis.

### 2.7. Tissue Distribution Experiments

Thirty rats were divided randomly into five groups and the rats were given single orally administration of PU-48 (12 mg/kg, 1.2 mg/mL in the SNEDDS formulation). After the administration, blood samples (1.5 mL) were drawn from the ophthalmic venous plexus at 0.25, 1, 6, 12, and 24 h. After the final time point, the rats were sacrificed and thirteen tissues (liver, spleen, lung, kidney, heart, muscle, colon, intestine, stomach, bladder, testicle, adipose, brain) were collected. Tissues samples were rinsed with ice-cold 0.9% normal saline and blotted dry with filter paper. All the samples were weighted and stored at −20 °C until analysis.

### 2.8. Excretion Experiments

Six male rats were given single orally administration of PU-48 (12 mg/kg, 1.2 mg/mL in the SNEDDS formulation) and housed in separate stainless-steel metabolism cages. Both feces and urine samples were collected at 0–1, 1–2, 2–4, 4–6, 6–8, 8–12, 12–24, 24–48, 48–72 and 72–96 h. In a separate study, bile samples were collected 0–1, 1–2, 2–4, 4–6, 6–8, 8–12, 12–24, 24–48, 48–72 and 72–96 h after oral administration of PU-48 (12 mg/kg, 1.2 mg/mL in the SNEDDS formulation) to six bile duct-cannulated rats.

### 2.9. Plasma Protein Binding Assay

The binding ratio of PU-48 to plasma protein was determined by equilibrium dialysis. A volume of 1 mL of plasma with PU-48 (0.25, 1 and 4 µg/mL) was added to a semi-permeable membrane bag (plasma chamber), the bag was place into the other clean tube with 9 mL PBS (buffer chamber). The equilibrium dialysis was performed at 37 °C for 24 h. At the end of dialysis, 500 μL each of post-dialysis samples from the plasma and the buffer chambers were collected and analyzed by HPLC method. The plasma protein binding of PU-48 was calculated from the concentration of PU-48 in PBS (free concentration) and in plasma (free + bound) according to equation: Bound (%) = (Conc_plasma chamber_ − Conc_buffer chamber_)/Conc_plasma chamber_ × 100%.

### 2.10. Pharmacokinetic Parameters and Statistical Analysis

The non-compartmental analysis method was used to calculate the pharmacokinetic parameters of PU-48 using Drug and Statistical Version 3.0 (DAS 3.0) software (the Mathematical Pharmacology Committee, Chinese Pharmacological Society, Beijing, China). The maximum peak concentration of the drug in plasma (*C*_max_) and the time to reach the maximum concentration (*T*_max_) were obtained directly from the experimental data. The area under the plasma concentration-time curves from 0 to infinity (i.e., AUC_0–∞_) and from 0 to the time of the last quantifiable concentration (AUC_0–t_) was calculated by the trapezoidal summation. The terminal elimination rate constant (*K*e) was derived from the slope of the linear regression curve by fitting the natural logarithms of the terminal concentrations versus time. The terminal elimination half-life (*t*_1/2_) was calculated by 0.693/*K*e. The data from the quality control (QC) samples were examined by a one-way analysis of variance (ANOVA). All values were expressed as mean ± SD (Standard Deviation).

## 3. Results

### 3.1. Method Validation

There was no significant interfering peak observed from endogenous substances in the biological samples at the retention time of PU-48 and IS. The typical chromatograms of PU-48 and IS (in plasma, urine, bile, liver, muscle, and adipose, respectively) are detected by LC-MS/MS system and presented in Supplementary Materials Figures S1–S6. Calibration curve of PU-48 in the biological samples showed linearity at the concentration ranging from 0.1 to 1000 ng/mL with correlation coefficient (*r*^2^) exceeding 0.99. The intra- and inter-day precisions and accuracy of analysis (error %) were less than 9.37% and 13.41%, respectively. The mean extraction recovery of PU-48 was greater than 86.5%. The HPLC method for quantification PU-48 in rat feces and plasma were showed in Supplementary Materials Figures S7 and S8.

### 3.2. Pharmacokinetic Parameters

**Figure 2:**
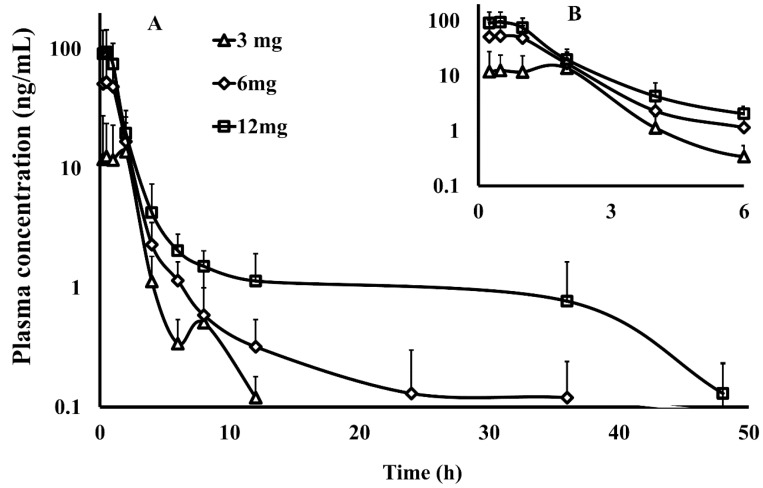
The plasma concentration–time curve within 48 h ((**A**), the main graph) and 6 h ((**B**), the inset) by semi-log data plot (the y-coordinate was logarithmic coordinates) of methyl 3-amino-6-methoxythieno [2,3-b] quinolone-2-carboxylate (PU-48) after a single oral administration of PU-48 self-nanomicroemulsifying drug delivery system (SNEDDS) at three doses (3, 6, 12 mg/kg) in rats detected by the liquid chromatography-tandem mass spectrometry (LC-MS/MS) method (*n* = 6, mean ± SD).

The validated method was applied to the determination of PU-48 concentration in plasma after a single oral administration at the three doses of 3, 6 and 12 mg/kg of PU-48 SNEDDS in rats (*n* = 6). The mean plasma concentration—time curves of PU-48 are shown in Figure 2. The relevant pharmacokinetic parameters of PU-48 were analyzed by non-compartmental model and shown in Table 1 as follows: The peak plasma concentrations (*C*_max_) were 12.6 ± 11.1, 52.9 ± 46.8, 94.3 ± 49.6 ng/mL; the time to reach the maximum concentration (*T*_max_) were 1.2 ± 0.9, 0.6 ± 0.3, 0.5 ± 0.3 h; the area under the curve (AUC_0–∞_) were 60.5 ± 38.5, 108.2 ± 52.5, 180.7 ± 62.5 ng·h/mL; and the plasma half-life (*t*_1/2_) were 7.1 ± 2.9, 7.0 ± 2.7, and 6.9 ± 3.5 h, respectively.

These results showed that PU-48 had a rapid absorption, could be detected at first time point (0.25 h), and reached the peak plasma concentration at the second time point (0.5 h) after oral administration of 3, 6, and 12 mg/kg, respectively. The data showed that the pharmacokinetic parameters of PU-48 (*C*_max_ and AUC_0–t_) were increased by the dose-dependent manner within the range from 3 mg/kg to 12 mg/kg in rat plasma with no significant difference in *t*_1/2_ and *T*_max_ (*p* = 0.05, ANOVA). These findings demonstrated that the analysis method was suitable for the quantitative determination of PU-48 in rat plasma. Then, the plasma concentrations were rapidly decreased, and at the 12 h time point, low concentrations of PU-48 were quantifiable in plasma (0.1 ± 0.1, 0.3 ± 0.2 and 1.1 ± 0.8 ng/mL, respectively). After oral administration at the 48 h time point, the mean plasma concentration were dropped below the LLOQ (See Supplementary Materials Table S7).

### 3.3. Tissue Distribution 

**Figure 3:**
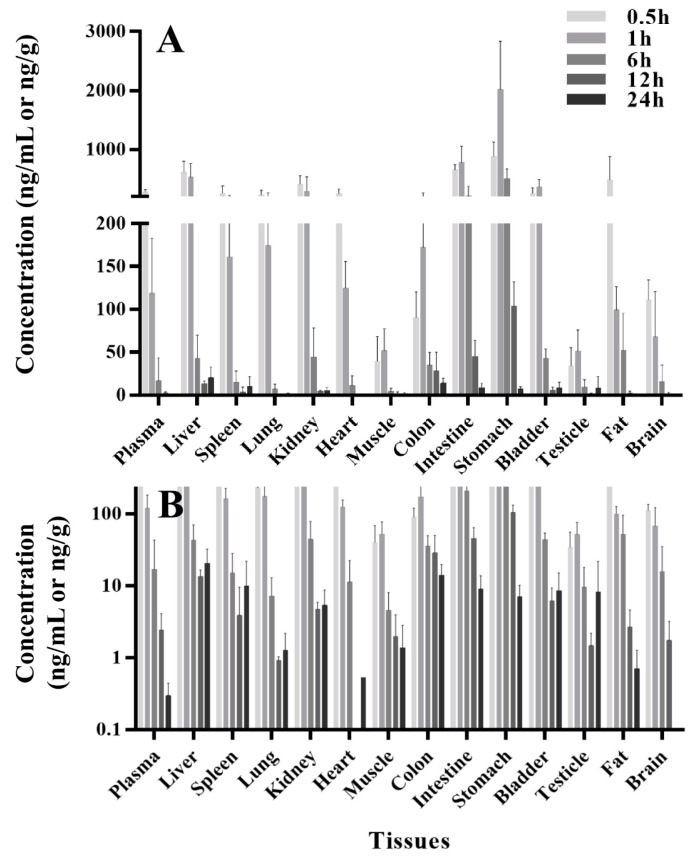
The concentrations-time course (**A**) and semilogdataplot (the y-coordinate was logarithmic coordinates, (**B**)) of methyl 3-amino-6-methoxythieno [2,3-b] quinolone-2-carboxylate (PU-48) in rat various tissues and plasma at 0.5, 1, 6, 12 and 24 h following single oral administration of PU-48 SNEDDS (12 mg/kg, *n* = 6).

The concentration-time profiles of thirteen tissues and plasma at same time point after a single oral administration of PU-48 SNEDDS (12 mg/kg) in rats were summarized in Table 2 and shown in Figure 3. Following oral administration, there was a rapid and extensive distribution of PU-48 in various tissues in rats. The peak time (*T*_max_) of PU-48 in analyzed tissues reached the maximum concentrations around 0.5 to 1 h, and the highest tissue peak concentration (*C*_max_) was observed in stomach (2017.8 ± 821.5 ng/g), followed by small intestine (777.4 ± 277.3 ng/g), liver (622.2 ± 182.8 ng/g), kidney (415.8 ± 137.7 ng/g) and bladder (367.7 ± 123.8 ng/g), indicating that PU-48 could be absorbed and entered into liver. The distributed maximum concentrations of PU-48 in these tissues were higher than that in the plasma (276.7 ± 42.4 ng/mL). The concentrations of PU-48 in most tissues were decreased at 6 h, and were lower than 10 ng/g except liver and colon at 24 h. Moreover, no retention or accumulation was observed in these tissues. The peak concentration of PU-48 in brain (111.1 ± 23.3 ng/mL) suggested that it could cross the blood brain barrier. The present results showed higher concentration and faster distribution of PU-48 into kidney and bladder, suggesting that PU-48 could rapidly reached its target tissues to produce diuretic effect.

### 3.4. Excretion

**Figure 4:**
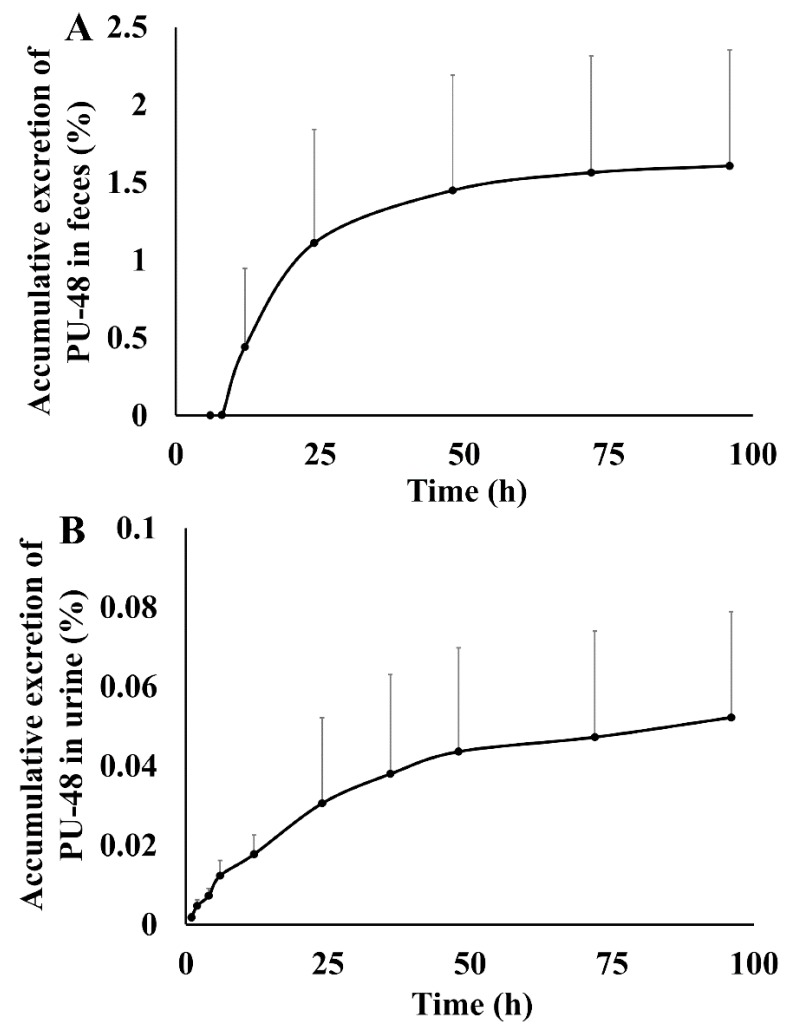

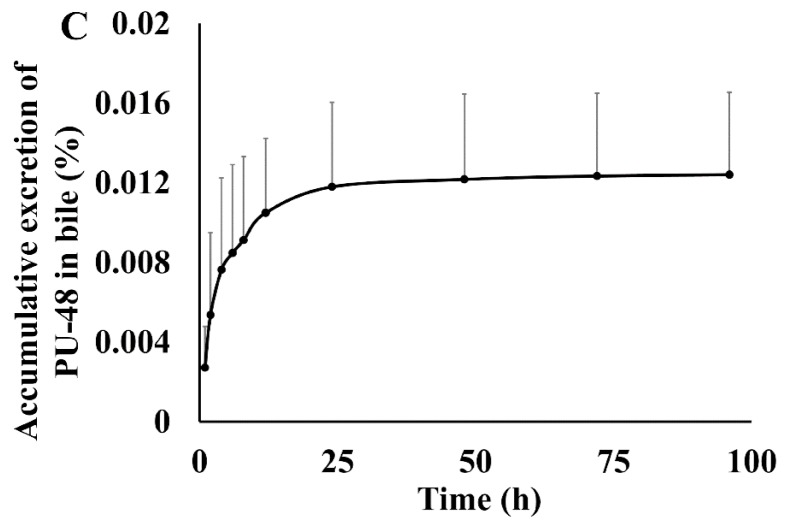
Feces, urinary and bile cumulative excretion profile of methyl 3-amino-6-methoxythieno [2,3-b] quinolone-2-carboxylate (PU-48) after single oral dose of 12 mg/kg in rats (*n* = 6).

After oral administration of PU-48 SNEDDS (12 mg/kg) in rats, the accumulatively excreted amounts of PU-48 in urine, feces, and bile were shown in Figure 4. The cumulative excretion ratios of PU-48 were 1.60 ± 0.74% in feces within 96 h, 0.052 ± 0.0267% in urine within 96 h, and 0.0124 ± 0.004% in bile within 96 h, respectively. The total excretion of PU-48 parent drug in the urine, feces, and bile was less than 2%. This suggests that the major route of elimination of PU-48 is via metabolic clearance (See Supplementary Materials Tables S8–S10).

### 3.5. Plasma Protein Binding

The results of in vitro plasma protein binding were shown in Table 3. In the range of PU-48 concentrations (0.25, 1 and 4 µg/mL), there were higher ratios of PU-48 plasma protein binding in both rat (90.70 ± 2.18%, 91.06 ± 0.78% and 90.83 ± 1.17%, respectively), and in human (91.60 ± 1.57%, 91.48 ± 0.64% and 89.90 ± 1.50%, respectively). No species and PU-48 concentration difference was observed in plasma binding.

## 4. Discussion

The renal transport proteins have been the important targets for the design of diuretic drugs. Several classes of inhibitors of electrolytic transport including sodium-potassium-chlorine co-transport (furosemide), or sodium-chlorine co-transport (hydrochlorothiazide) have been used in clinic for treatment of edema, heart failure and hypertension [26,27]. However, their adverse reactions such as electrolyte disturbance and dysglycemia have limited effectively marketing the conventional diuretics [28]. It is necessary to develop new diuretic drugs with improved drug interference electrolytic balance properties.

The inhibitor of urea transporter (UT), different from the electrolytic transport blockers, has unique diuretic effect because of their salt-sparing mechanism [29,30]. As a selective inhibitor of UT-A isoform, PU-48 exhibited potent diuretic activity in both wild-type and UT-gene knockout mice, suggesting PU-48 has the potential to become a novel diuretic drug. To the best of our knowledge, the present study is the first study discussing the pharmacokinetic properties of PU-48, including the absorption, tissue distribution and excretion.

As a first step of this research, the rapid, specific and sensitive analysis methods were developed and validated for the quantitative determination of PU-48 in the biosamples, including LC-MS/MS method for rat plasma, thirteen tissues, urine, and bile, respectively; and HPLC method for rat feces, and plasma protein binding of both rat and human. The present results showed that the LC-MS/MS method was validated with a dynamic calibration range from 0.1 to 1000 ng/mL, with a short run time of 8 min, and with isocratic elution. These findings demonstrated that the analysis methods were suitable for the quantitative determination of PU-48 in the biosamples and could meet the requirements for the pharmacokinetic research of the thienoquinolin derivate PU-48 [25,31]. 

After oral three doses of 3, 6, and 12 mg/kg PU-48 SNEDDS formulation, the plasma concentration-time courses and pharmacokinetic parameters of PU-48 showed a rapid absorption and elimination by the dose-dependent manner in rats. The results showed that PU-48 could be detected at first time point (0.25 h) and reached the peak plasma concentration (*T*_max_) at the second time point (0.5 h) after oral administration. Both the *C*_max_ and AUC_0–t_ values of PU-48 were increased by the dose-dependent and linear manner within the range from 3 mg/kg to 12 mg/kg in rat plasma. Moreover, the marked different of plasma half-life (*t*_1/2_) values were not observed. Similar to the previous other research, the present results suggested the pharmacokinetic profile of PU-48 prototype compound was first-order elimination kinetic characteristics within the oral three doses range in rat plasma [32,33,34,35]. After oral administration at the 48 h time point, the mean plasma concentration were dropped below the lower limit of quantitation (LLOQ), which suggested that PU-48 had a rapid and complete clearance in rat plasma. These results were also alike to other reported non-accumulative disposition in plasma in vivo [36,37]. However, because of oral study provides only limited information on disposition profiles of PU-48 compound. Therefore, further investigation into the pharmacokinetic parameters, such as absolute bioavailability by intravenous administration in the future, is required.

PU-48 can be rapidly and extensively distributed in various tissues in rats. The peak time (*T*_max_) of PU-48 in analyzed tissues reached at 0.5 to 1 h, and the highest tissue peak concentration (*C*_max_) was observed in stomach, followed by small intestine, liver, kidney, and bladder; suggesting that PU-48 was mainly absorbed via the gastrointestinal tract and was a moderate absorption in liver. The distributed maximum concentrations of PU-48 in these tissues were higher than that in the plasma at the same time point after a single oral administration of PU-48 SNEDDS (12 mg/kg) in rats. The concentrations of PU-48 in most tissues were decreased at 6 h, and were lower than that 10 ng/g except liver and colon at 24 h. Similar to the several reported pharmacokinetic studies recently, the present results suggested that the tissue distribution of PU-48 was reversible and non-accumulations were observed in these tissues [38,39,40].

It is worth to note that our results showed that PU-48 could cross the blood brain barrier (BBB) and was rapidly distributed into the brain tissue. This is different from classical electrolyte transport inhibitors, such as salt-sparing diuretic action (urearetics) [41,42]. Thus, PU-48 might be beneficial for treatment of cerebral edema in clinic [43,44]. Furthermore, the present results showed that the higher concentration and faster distribution of PU-48 into kidney and bladder, suggesting that PU-48 could rapidly reached the urinary system tissues to produce diuretic effect.

It has been accepted that the extensive elimination of a drug contains two processes—metabolism and excretion—which major performed by metabolic enzymes and transporters in liver, kidney, and small intestine, respectively [45,46,47,48]. In the present research, the standard of metabolites was not available. Therefore, only the parent drug of PU-48 was measured and quantitated. After oral administration of PU-48 SNEDDS (12 mg/kg) in rats, the cumulative excretion ratio of PU-48 was 1.60% in feces, 0.052% in urine, and 0.0124% in bile, within 96 h, respectively. The total excretion of PU-48 parent drug in the urine, feces and bile was less than 2%, suggesting that the major route of elimination of PU-48 might be via metabolic clearance.

Because the kidneys play an important role in drug metabolism and excretory, and it is the target organ by PU-48 diuretic role. Therefore, understanding the pharmacokinetic and pharmacodynamic (PK-PD) relationship of PU-48, and its underlining mechanism, is critical [49,50,51,52]. In the present research, low recoveries of PU-48 parent drug indicated that PU-48 had an almost complete metabolism in rats. Further researches to identify the metabolic pathways of PU-48 are warranted. In addition, it has been reported that the plasma protein binding is another way of drug temporary elimination from plasma [53]. The present results suggested that the plasma protein binding of PU-48 was more than 90% in rats and human, and plasma protein binding appeared to be concentration and species independent.

## 5. Conclusions

The present research is the first report of pharmacokinetics of PU-48, a novel diuretic urea transporter inhibitor in rats. After three oral doses of PU-48 SNEDDS formulation, the plasma concentration-time courses and pharmacokinetic parameters (such as *C*_max_, *T*_max_ and AUC_0–t_) of PU-48 showed a rapid absorption and elimination in rats with a dose-dependent and linear manner. Following oral administration, PU-48 was rapidly and extensive distributed into thirteen tissues, and the higher concentration were observed in alimentary tract, liver, kidney, and bladder, than in plasma at same time point; suggesting that PU-48 could rapidly reached its target tissues to produce diuretic effect. The concentrations of PU-48 in most tissues were significant decreased at 6 h time point, and no accumulation at 24 h. The present results showed that the total accumulative excretions of PU-48 parent drug were less than 2% from the urine, feces and bile, suggesting that the major route of elimination of PU-48 might be via metabolic clearance. Therefore, the study on the potential metabolites of PU-48 is needed to investigate after oral administration. Plasma protein binding PU-48 were high and no species difference was observed in rat and human. This research is the first report on the preclinical pharmacokinetic properties of PU-48, and it provides important disposition information for further development of PU-48 as the unique salt-sparing diuretic drug candidate.

## Figures and Tables

**Table 1:** Pharmacokinetic parameters of methyl 3-amino-6-methoxythieno [2,3-b] quinolone-2-carboxylate (PU-48) after single oral administration of PU-48 SNEDDS at the doses of 3, 6 and 12 mg/kg in rats plasma (mean ± SD, *n* = 6).

Pharmacokinetic Parameters	Unit	PU-48		
		3 mg/kg	6 mg/kg	12 mg/kg
*C*_max_	ng/mL	12.6 ± 11.1	52.9 ± 46.8	94.3 ± 49.6
*T*_max_	h	1.19 ± 0.94	0.63 ± 0.31	0.50 ± 0.27
AUC_0–t_	ng·h/mL	60.0 ± 38.3	106.8 ± 53.0	178.9 ± 60.1
AUC_0–__∞_	ng·h/mL	60.5 ± 38.5	108.2 ± 52.5	180.7 ± 62.5
*t*_1/2_	h	7.14 ± 2.93	7.00 ± 2.70	6.87 ± 3.49

*C*_max_, maximum concentration; *T*_max_, peak time; *t*_1/2_, half life time; AUC_0–t_, area under curve from time zero to the time of last quantifiable concentration; AUC_0–∞_, area under curve from time zero to infinity.

**Table 2:** Tissue distribution-time course after single oral administration of methyl 3-amino-6-methoxythieno [2,3-b] quinolone-2-carboxylate (PU-48) SNEDDS (12 mg/kg) in rats (*n* = 6, mean ± SD).

Tissues	Concentration of PU-48 (ng/mL or ng/g)				
	0.5 h	1 h	6 h	12 h	24 h
Plasma	276.7 ± 42.4	119.1 ± 63.5	16.8 ± 26.4	2.4 ± 1.7	0.3 ± 0.1
Liver	622.2 ± 182.8	534.3 ± 228.4	42.7 ± 27.3	13.4 ± 3.1	20.4 ± 12.2
Spleen	265.9 ± 120.1	161.2 ± 64.0	15.2 ± 12.9	3.8 ± 5.7	9.9 ± 12.0
Lung	231.0 ± 79.3	174.3 ± 91.4	7.2 ± 5.7	0.9 ± 0.1	1.3 ± 0.9
Kidney	415.8 ± 137.7	296.6 ± 241.4	44.5 ± 33.9	4.7 ± 1.1	5.3 ± 3.4
Heart	251.1 ± 78.4	124.6 ± 31.0	11.3 ± 11.2	ND	0.5
Muscle	39.8 ± 28.6	52.0 ± 25.5	4.5 ± 3.5	2.0 ± 1.9	1.4 ± 1.5
Colon	90.4 ± 30.0	172.5 ± 89.5	35.2 ± 14.7	28.6 ± 21.7	14.1 ± 5.6
Intestine	654.8 ± 89.3	777.4 ± 277.3	209.3 ± 166.5	45.4 ± 18.9	9.0 ± 4.7
Stomach	887.3 ± 234.9	2017.8 ± 821.5	508.8 ± 160.5	104.0 ± 28.4	7.0 ± 3.1
Bladder	258.2 ± 94.2	367.7 ± 123.8	43.4 ± 10.7	6.1 ± 3.2	8.6 ± 6.5
Testicle	34.2 ± 21.2	51.7 ± 24.4	9.5 ± 8.5	1.5 ± 0.7	1.4 ± 1.1
Fat	480.6 ± 398.1	99.3 ± 26.9	52.3 ± 43.2	2.7 ± 1.9	0.7 ± 0.6
Brain	111.1 ± 23.3	68.1 ± 52.7	15.7 ± 19.3	1.7 ± 1.5	ND

ND: not detected.

**Table 3:** Determination of the protein binding ratio of methyl 3-amino-6-methoxythieno [2,3-b] quinolone-2-carboxylate (PU-48) in rat and human plasma (*n* = 5).

Species	Added Concentration (µg/mL)	Concentration in Plasma (µg/mL)	Concentration in Buffer (µg/mL)	Protein-Binding Ratio (%)
Rat	0.25	0.127 ± 0.007	0.012 ± 0.003	90.70 ± 2.18
	1	0.524 ± 0.032	0.047 ± 0.002	91.06 ± 0.78
	4	2.128 ± 0.166	0.194 ± 0.019	90.83 ± 1.17
Human	0.25	0.146 ± 0.008	0.012 ± 0.003	91.60 ± 1.57
	1	0.611 ± 0.027	0.052 ± 0.003	91.48 ± 0.64
	4	2.290 ± 0.093	0.231 ± 0.029	89.90 ± 1.50

## Supplementary Materials

Supplementary Materials are available online at http://www.mdpi.com/1999-4923/10/3/124/s1, Figure S1: Representative multiple reaction monitoring (MRM) chromatograms of (A) blank plasma; (B) blank plasma spiked with methyl 3-amino-6-methoxythieno [2,3-b] quinolone-2-carboxylate (PU-48, 10 ng/mL), and internal standard (IS, 500 ng/mL); and (C) a rat plasma sample at 0.5 h after a single oral administration of PU-48 (12 mg/kg), Figure S2: Representative MRM chromatograms of (A) blank urine; (B) blank urine spiked with methyl 3-amino-6-methoxythieno [2,3-b] quinolone-2-carboxylate (PU-48, 10 ng/mL) and internal standard (IS, 500 ng/mL); and (C) a rat urine sample at 24 h after a single oral administration of PU-48 (12 mg/kg), Figure S3: Representative MRM chromatograms of (A) blank bile; (B) blank bile spiked with methyl 3-amino-6-methoxythieno [2,3-b] quinolone-2-carboxylate (PU-48, 10 ng/mL) and internal standard (IS, 500 ng/mL); and (C) a rat bile sample at 1 h after a single oral administration of PU-48 (12 mg/kg), Figure S4: Representative MRM chromatograms of (A) blank liver; (B) blank liver spiked with methyl 3-amino-6-methoxythieno [2,3-b] quinolone-2-carboxylate (PU-48, 10 ng/mL) and internal standard (IS, 500 ng/mL); and (C) a rat liver sample at 1 h after a single oral administration of PU-48 (12 mg/kg), Figure S5: Representative MRM chromatograms of (A) blank muscle; (B) blank muscle spiked with methyl 3-amino-6-methoxythieno [2,3-b] quinolone-2-carboxylate (PU-48, 10 ng/mL) and internal standard (IS, 500 ng/mL); and (C) a rat muscle sample at 1 h after a single oral administration of PU-48 (12 mg/kg), Figure S6: Representative multiple reaction monitoring (MRM) chromatograms of (A) blank adipose; (B) blank adipose spiked with methyl 3-amino-6-methoxythieno [2,3-b] quinolone-2-carboxylate (PU-48, 10 ng/mL) and internal standard (IS, 500 ng/mL); and (C) a rat adipose sample at 1 h after a single oral administration of PU-48 (12 mg/kg), Figure S7: Representative HPLC-UV chromatograms of (A) blank feces; (B) blank feces spiked with methyl 3-amino-6-methoxythieno [2,3-b] quinolone-2-carboxylate (PU-48, 0.02 µg/mL) and internal standard (IS, 0.2 µg/mL); and (C) a rat feces sample at 24 h after a single oral administration of PU-48 (12 mg/kg), Figure S8: Representative HPLC-UV chromatograms of (A) blank plasma; (B) blank plasma spiked with methyl 3-amino-6-methoxythieno [2,3-b] quinolone-2-carboxylate (PU-48, 0.2 µg/mL) and internal standard (IS, 0.2 µg/mL); and (C) a rat plasma sample at 0.25 h after a single oral administration of PU-48 (12 mg/kg), Table S1: Calibration curve correlation coefficient and linear range of methyl 3-amino-6-methoxythieno [2,3-b] quinoline-2-carboxylate (PU-48) in biological samples were detected by LC-MS/MS method (*n* = 3), Table S2: Precision and accuracy data for methyl 3-amino-6-methoxythieno [2,3-b] quinoline-2-carboxylate (PU-48) in biological samples were detected by LC-MS/MS method (intra-day: *n* = 5; inter-day: *n* = 5 series per day, 3 days), Table S3: Extraction recovery of methyl 3-amino-6-methoxythieno [2,3-b] quinoline-2-carboxylate (PU-48) in biological samples detected by LC-MS/MS method (*n* = 3), Table S4: Ration of peak value between methyl 3-amino-6-methoxythieno [2,3-b] quinolone-2-carboxylate (PU-48) and megestrol acetate (internal standard, IS, 0.2 µg/mL) in blank rat plasma detected by HPLC method (*n* = 5), Table S5: Precision and accuracy data of methyl 3-amino-6-methoxythieno [2,3-b] quinolone-2-carboxylate (PU-48) detected by HPLC method in rat plasma (*n* = 5; series per day; 5 days), Table S6: Stability data of methyl 3-amino-6-methoxythieno [2,3-b] quinolone-2-carboxylate (PU-48) detected by HPLC method in rat plasma (Mean ± SD, *n* = 3), Table S7: Plasma concentration - time courses of methyl 3-amino-6-methoxythieno [2,3-b] quinoline-2-carboxylate (PU-48) after single oral administration of PU-48 SNEDDS at the dose of 3, 6 and 12 mg/kg evaluated by the validated LC-MS/MS method in rats (*n* = 6), Table S8: Mean urine accumulative excretion amount of methyl 3-amino-6-methoxythieno [2,3-b] quinoline-2-carboxylate (PU-48) after oral administration of PU-48 SNEDDS (12 mg/kg) in rats (*n* = 6, mean ± SD), Table S9: Mean feces accumulative excretion amount of methyl 3-amino-6-methoxythieno [2,3-b] quinoline-2-carboxylate (PU-48) after oral administration of PU-48 SNEDDS (12 mg/kg) in rats (*n* = 6, mean ± SD), Table S10: Mean bile accumulative excretion amount of methyl 3-amino-6-methoxythieno [2,3-b] quinoline-2-carboxylate (PU-48) after oral administration of PU-48 SNEDDS (12 mg/kg) in rats (*n* = 6, mean ± SD).
