# Supplementary material for: Pharmacokinetics, Tissue Distribution and Excretion of a Novel Diuretic (PU-48) in Rats
Source: Pharmaceutics. 2018 Aug 8;10(3):124. doi: 10.3390/pharmaceutics10030124 (PMC6160999; doi:10.3390/pharmaceutics10030124)
Supplement: Supplementary file 1 [file pharmaceutics-10-00124-s001.pdf]

Supplementary Materials: Pharmacokinetics, Tissue Distribution and Excretion of a Novel Diuretic (PU-48) in Rats

Zhi-Yuan Zhang, Hua Zhang, Dan Liu, Ying-Yuan Lu, Xin Wang, Pu Li, Ya-Qing Lou, Bao-Xue Yang, Ya-Xin Lou, Chuang Lu, Qiang Zhang and Guo-Liang Zhang

HPLC Analysis and Method Validation

The concentration of PU-48 prototype in biological sample (feces and plasma protein binding analysis) was determined using a validated high-performance liquid chromatographic (HPLC) method in our preliminary experiment. Briefly, the analysis was performed using a HPLC consistent of model 510 pump with a model 2487 ultraviolet detector, a model Rheodyne 7725 injector, and a column oven. The analytical conditions were as follows: the detection wavelength: 293 nm; analytical column: a reversed-phase Acclaim C18 (4.6 mm × 250 mm, 5.0 μm, Dionex); column temperature: 25 C; mobile phase: acetonitrile: distilled water at a ratio of 60: 40 (*v*/*v*); flow rate: 1 mL/min. In this study, the methods for the feces samples, were evaluated for specificity, linearity, precision, accuracy and recovery.

The intra- and inter-day precision and accuracy were determined by analyzing five replicates of QC samples (low, medium and high concentrations of 0.005, 0.08 and 1.6 μg/mL) at each QC level on the same day and on five consecutive days. The precision was expressed as the relative standard deviation (RSD) between the measured values and the targeted values. The pass criterion was set at <15%. The accuracy was expressed as relative error (RE) between the measured values and the targeted values, which was set at < ± 15% (for the low concentration, the acceptance criterion was <20%). The stability of PU-48 in rat plasma was determined by analyzing QC samples stored in different conditions, including room temperature and light irradiation for 24 h, post-preparation (the extracted samples were stored at 4°C for 96 h), three cycles of freeze and thaw (freezing at -20°C for 24 h and thawing at room temperature) and long-term stored at −20°C for 3 months. All of the QC samples for stability assessment were analyzed in triplicate.

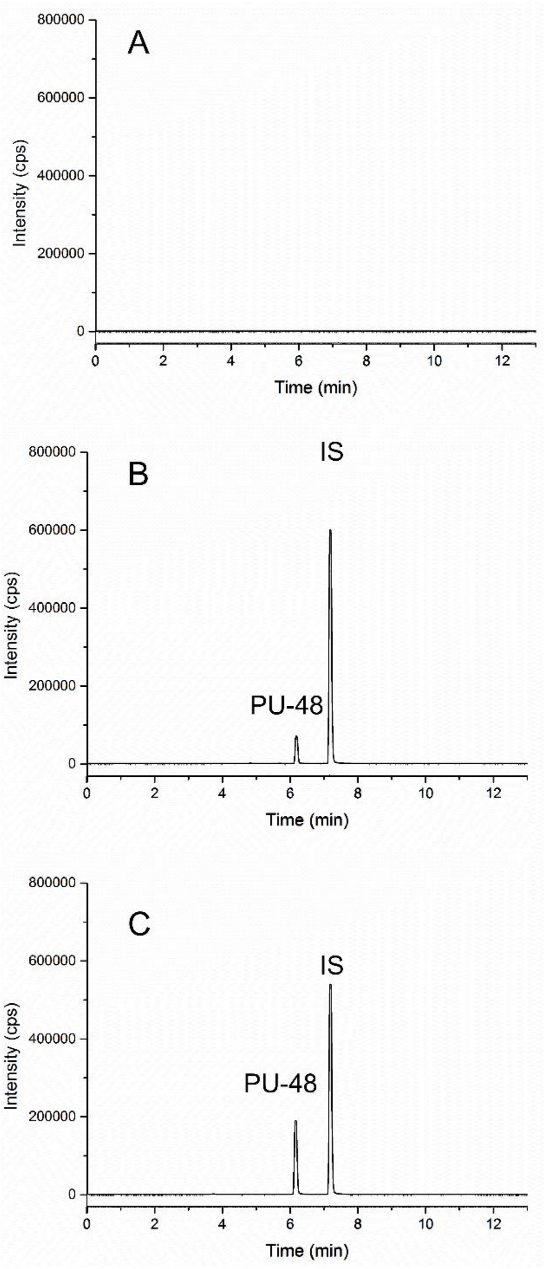

Figure S1. Representative multiple reaction monitoring (MRM) chromatograms of (**A**) blank plasma; (**B**) blank plasma spiked with methyl 3-amino-6-methoxythieno [2,3-b] quinolone-2- carboxylate (PU-48, 10 ng/mL) and internal standard (IS, 500 ng/mL) and (**C**) a rat plasma sample at 0.5 h after a single oral administration of PU-48 (12 mg/kg).

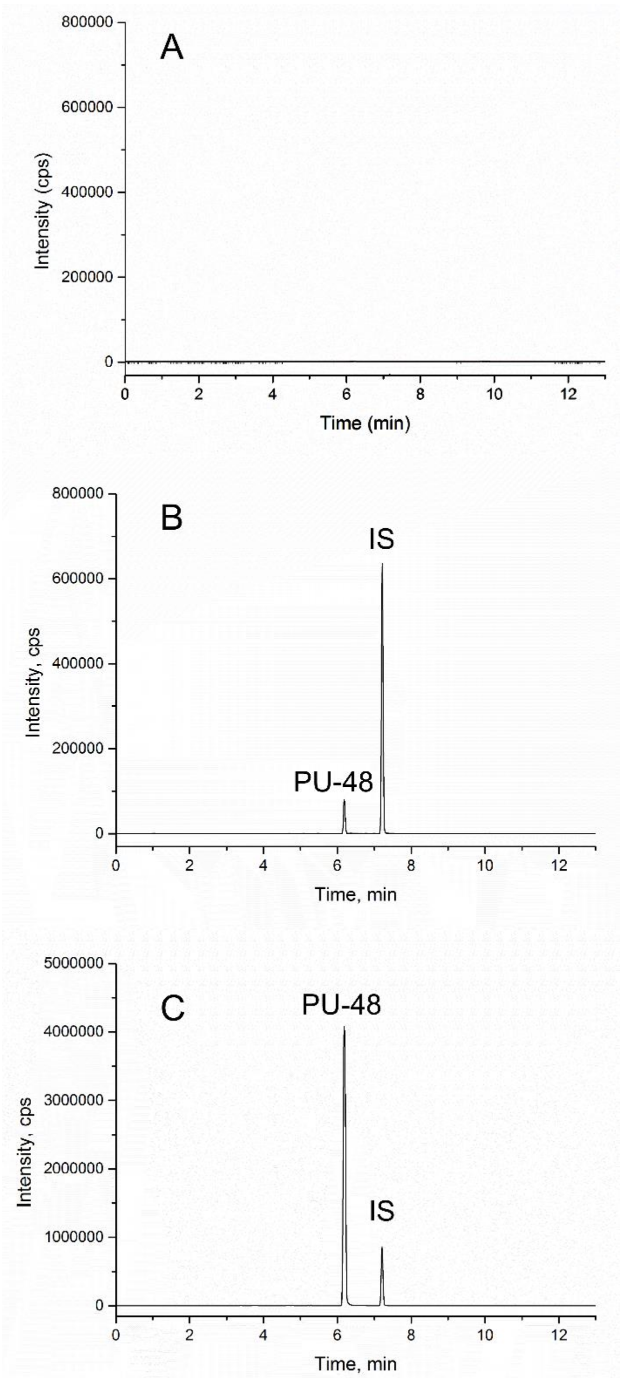

Figure S2. Representative multiple reaction monitoring (MRM) chromatograms of (**A**) blank urine; (**B**) blank urine spiked with methyl 3-amino-6-methoxythieno [2,3-b] quinolone-2- carboxylate (PU-48, 10 ng/mL) and internal standard (IS, 500 ng/mL) and (**C**) a rat urine sample at 24 h after a single oral administration of PU-48 (12 mg/kg).

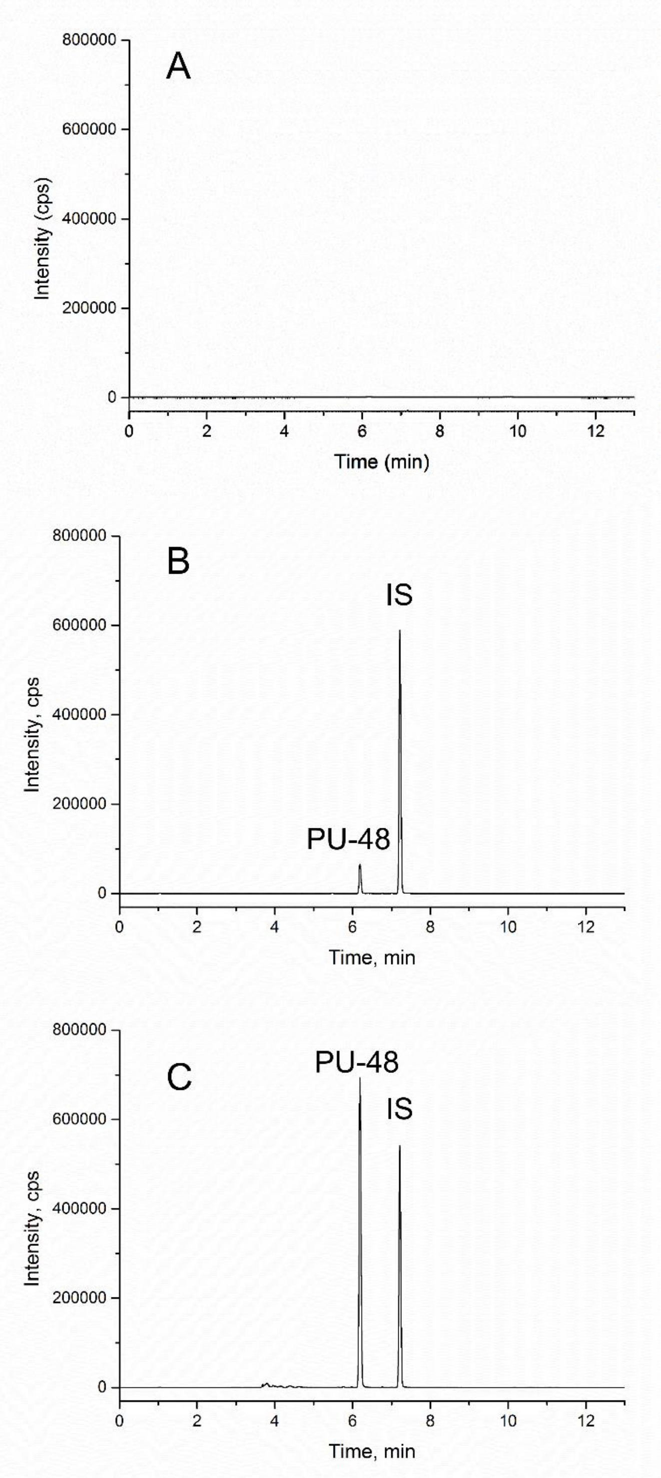

Figure S3. Representative multiple reaction monitoring (MRM) chromatograms of (**A**) blank bile; (**B**) blank bile spiked with methyl 3-amino-6-methoxythieno [2,3-b] quinolone-2-carboxylate (PU-48, 10 ng/mL) and internal standard (IS, 500 ng/mL) and (**C**) a rat bile sample at 1 h after a single oral administration of PU-48 (12 mg/kg).

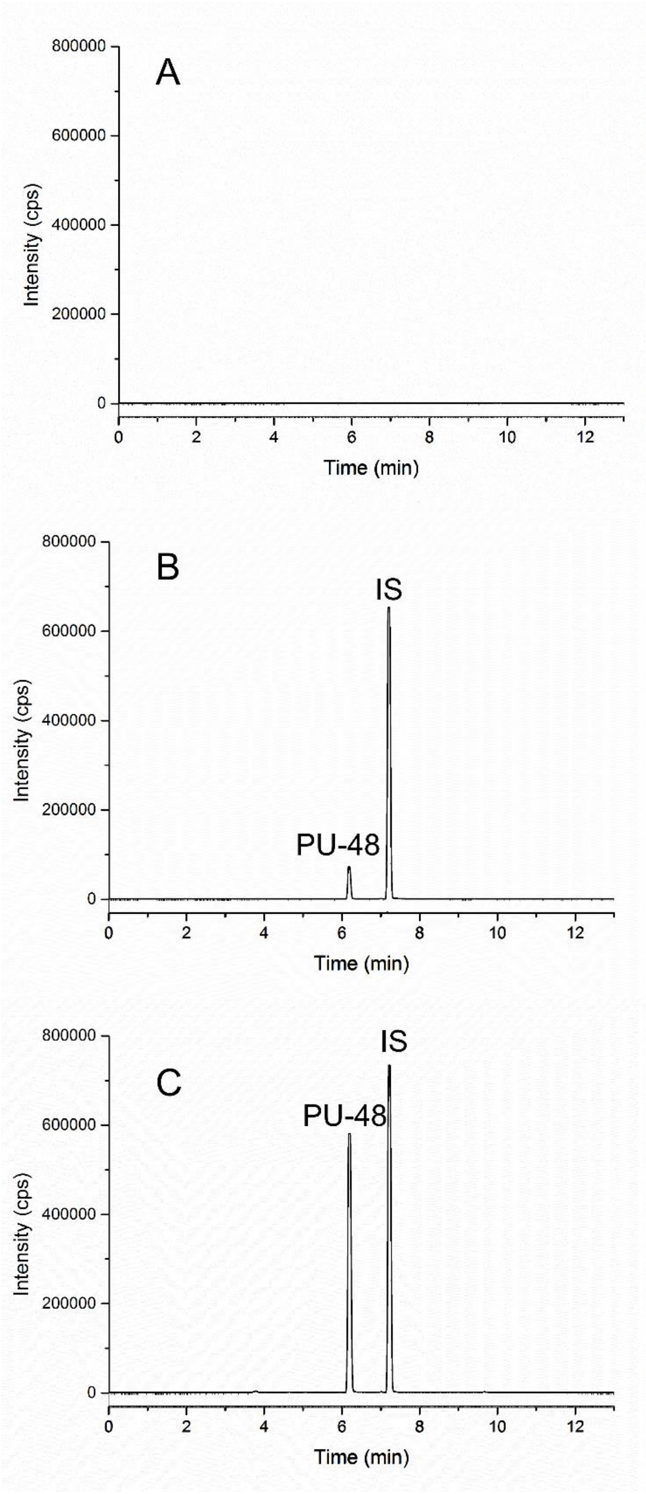

Figure S4. Representative multiple reaction monitoring (MRM) chromatograms of (**A**) blank liver; (**B**) blank liver spiked with methyl 3-amino-6-methoxythieno [2,3-b] quinolone-2- carboxylate (PU-48, 10 ng/mL) and internal standard (IS, 500 ng/mL) and (**C**) a rat liver sample at 1 h after a single oral administration of PU-48 (12 mg/kg).

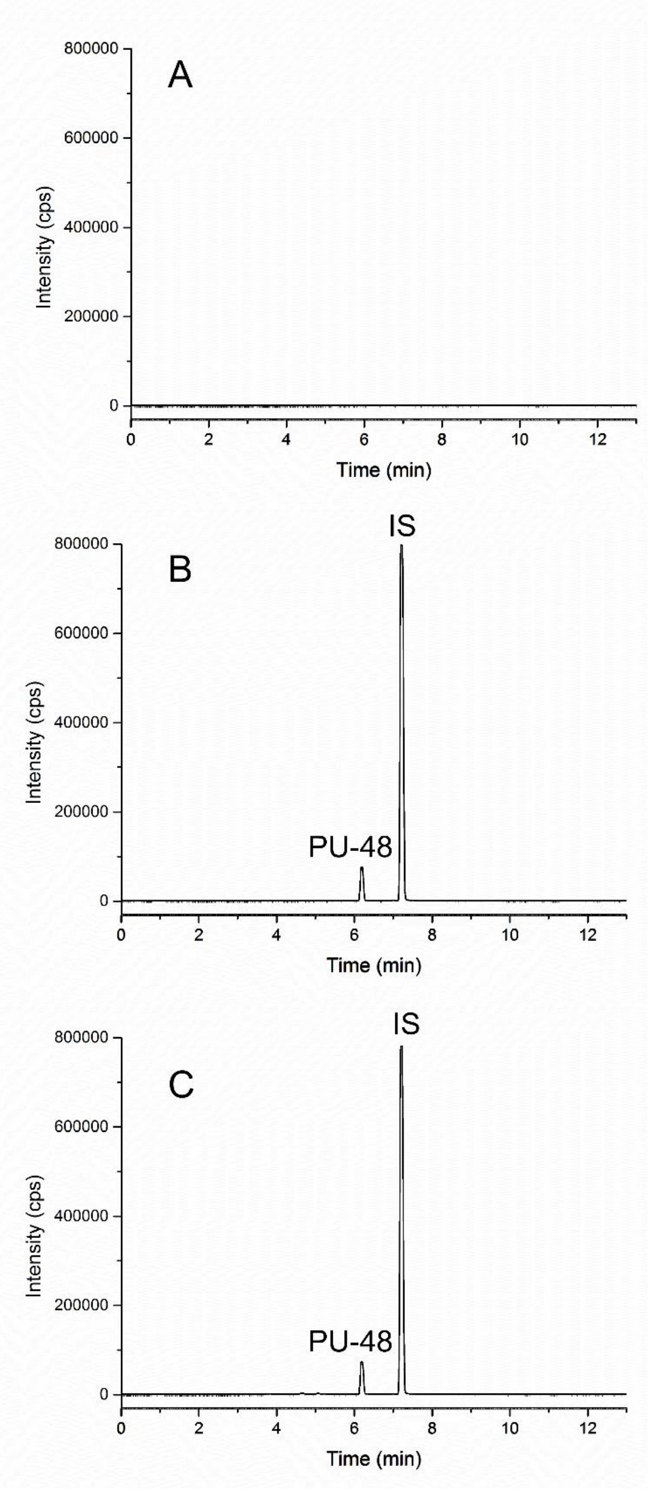

Figure S5. Representative multiple reaction monitoring (MRM) chromatograms of (**A**) blank muscle; (**B**) blank muscle spiked with methyl 3-amino-6-methoxythieno [2,3-b] quinolone-2- carboxylate (PU-48, 10 ng/mL) and internal standard (IS, 500 ng/mL) and (**C**) a rat muscle sample at 1 h after a single oral administration of PU-48 (12 mg/kg).

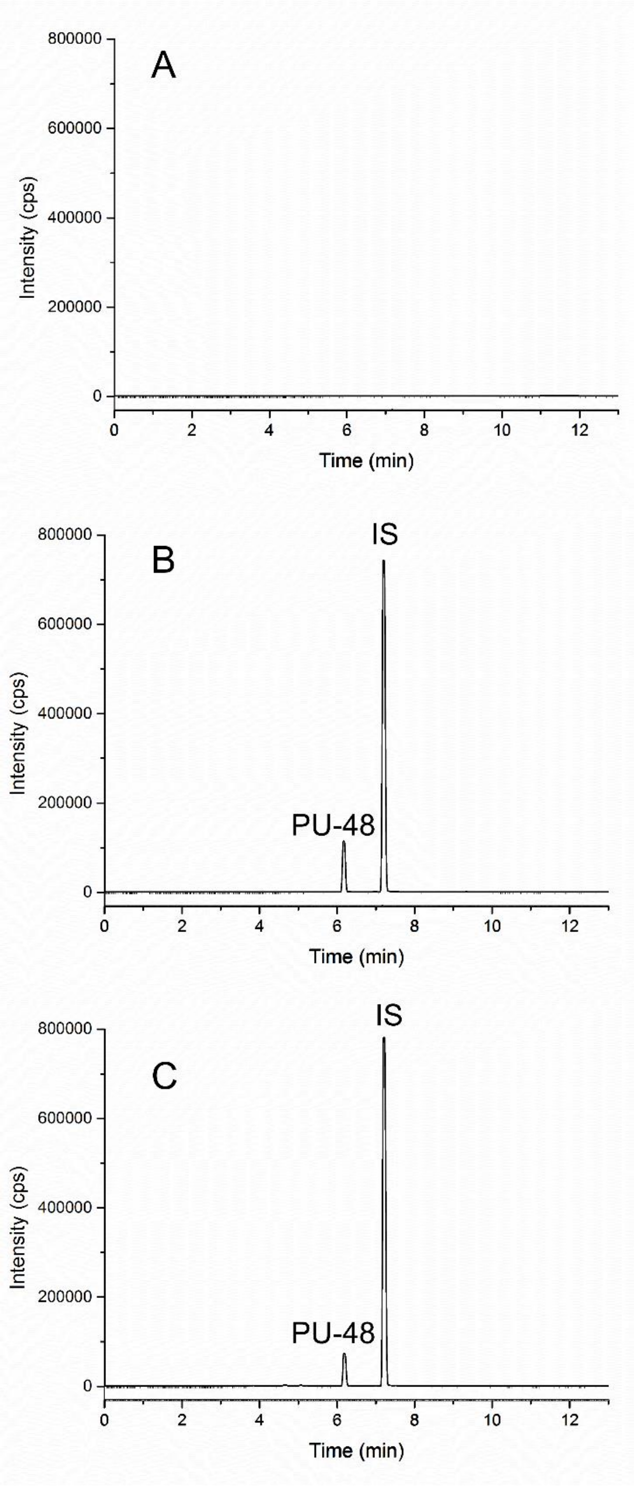

Figure S6. Representative multiple reaction monitoring (MRM) chromatograms of (**A**) blank adipose; (**B**) blank adipose spiked with methyl 3-amino-6-methoxythieno [2,3-b] quinolone-2- carboxylate (PU-48, 10 ng/mL) and internal standard (IS, 500 ng/mL) and (**C**) a rat adipose sample at 1 h after a single oral administration of PU-48 (12 mg/kg).

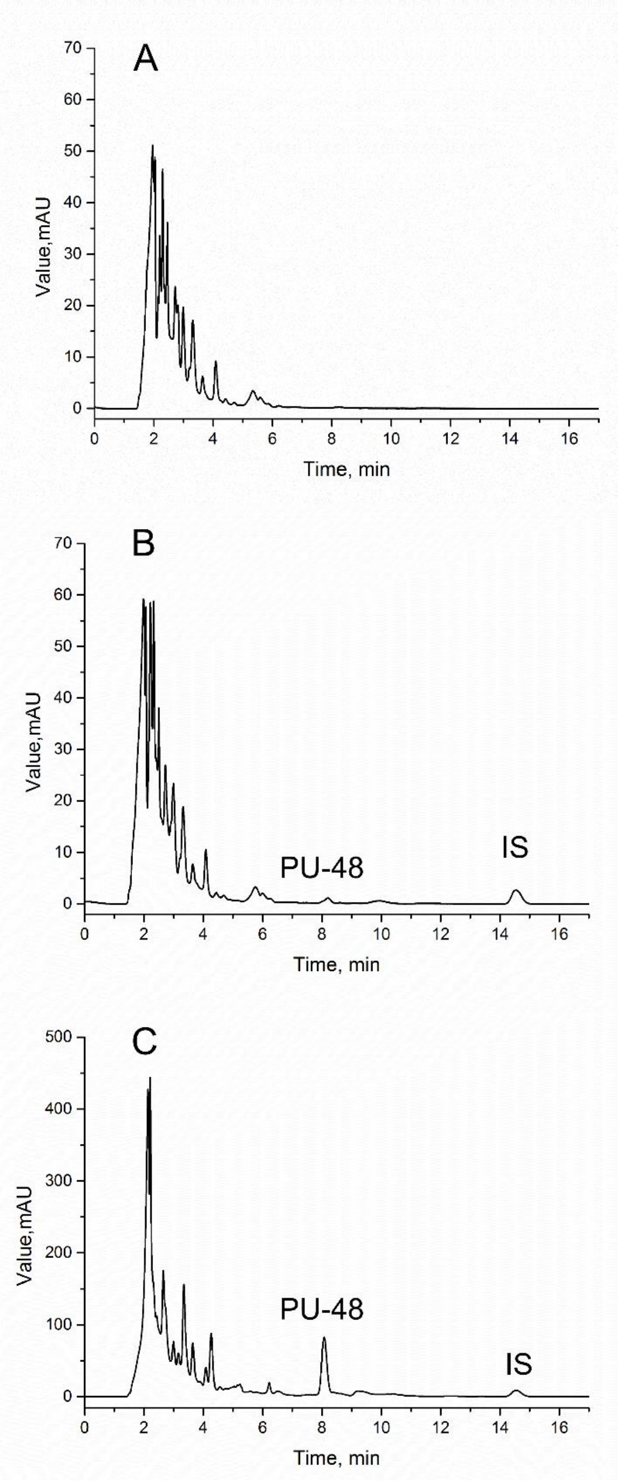

Figure S7. Representative HPLC-UV chromatograms of (**A**) blank feces; (**B**) blank feces spiked with methyl 3-amino-6-methoxythieno [2,3-b] quinolone-2-carboxylate (PU-48, 0.02 μg/mL) and internal standard (IS, 0.2 μg/mL) and (**C**) a rat feces sample at 24 h after a single oral administration of PU-48 (12 mg/kg).

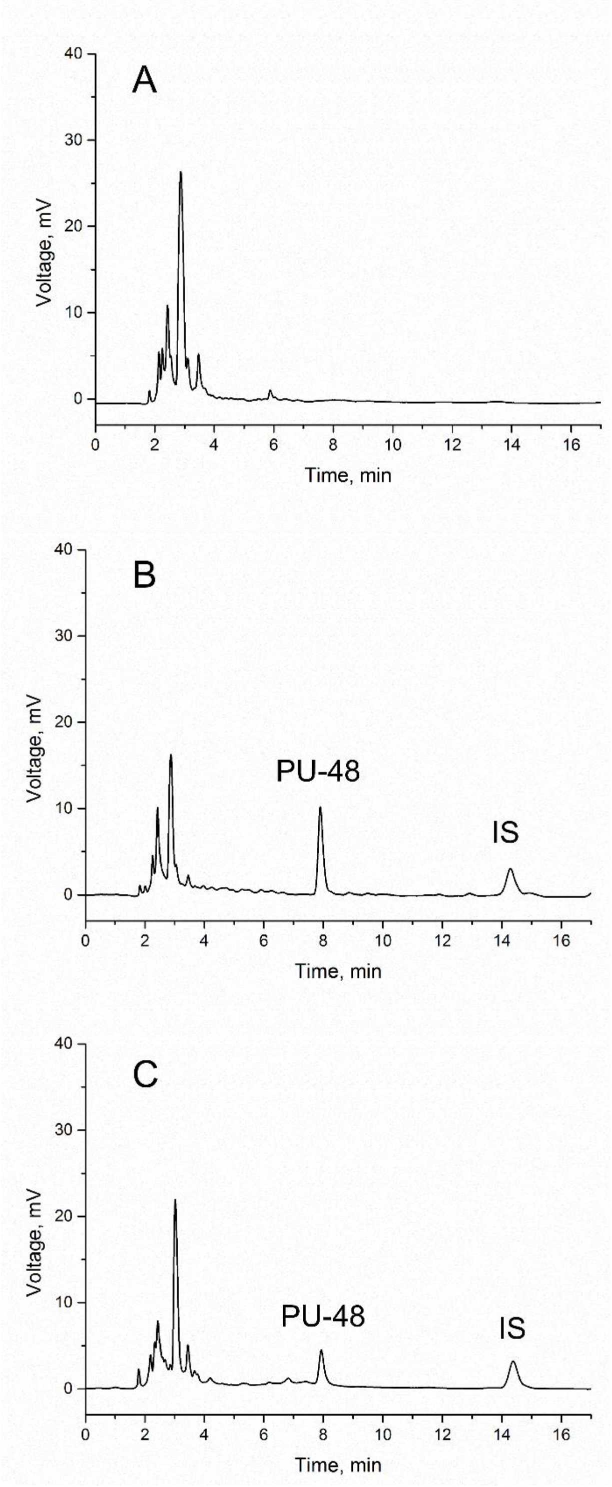

Figure S8. Representative HPLC-UV chromatograms of (**A**) blank plasma; (**B**) blank plasma spiked with methyl 3-amino-6-methoxythieno [2,3-b] quinolone-2- carboxylate (PU-48, 0.2 µg/mL) and internal standard (IS, 0.2 µg/mL) and (**C**) a rat plasma sample at 0.25 h after a single oral administration of PU-48 (12 mg/kg).

Table S1. Calibration curve correlation coefficient and linear range of methyl 3-amino-6-methoxythieno [2,3-b] quinoline-2-carboxylate (PU-48) in biological samples were detected by liquid chromatography-tandem mass spectrometry (LC-MS/MS) method (*n* = 3).

Biological Samples	Regression Equation	Correlation Coefficient (r^2)	Linear Range (ng/mL)
Plasma	$Y = 0.0072X + 0.0010$	0.9929	0.1–1000
Liver	$Y = 0.0067X + 0.0006$	0.9934	0.1–1000
Muscle	$Y = 0.0053X + 0.0011$	0.9901	0.1–1000
Fat	$Y = 0.0078X + 0.0006$	0.9937	0.1–1000
Urine	$Y = 0.0089X + 0.0010$	0.9944	0.1–1000
Feces	$Y = 0.0042X +0.0523$	0.9941	2–3200

The sample of feces was evaluated by high performance liquid chromatography (HPLC) method.

Table S2. Precision and accuracy data for methyl 3-amino-6-methoxythieno [2,3-b] quinoline-2-carboxylate (PU-48) in biological samples were detected by liquid chromatography-tandem mass spectrometry (LC-MS/MS) method (intra-day: n=5; inter-day: *n* = 5 series per day, 3 days).

Biological Samples	Nominal Concentrations (ng/mL)	Intra-Day		Inter-Day	
		Precision (RSD, %)	Accuracy (RE, %)	Precision (RSD, %)	Accuracy (RE, %)
Plasma	0.2	4.14	−0.37	5.49	−0.07
	10	2.29	12.95	8.29	1.88
	500	2.38	−9.55	3.19	−9.55
Liver	0.2	2.37	4.60	10.45	2.30
	10	4.92	7.35	5.33	4.43
	500	2.24	−11.37	3.36	−10.63
Muscle	0.2	10.33	1.36	9.75	−1.53
	10	10.16	−3.01	8.05	4.23
	500	4.40	−11.28	10.24	−2.02
Fat	0.2	5.23	−1.41	8.01	0.77
	10	2.83	9.27	5.14	7.32
	500	5.01	−7.85	4.84	−6.88
Urine	0.2	9.16	−2.94	7.82	−4.91
	10	6.66	6.77	4.80	9.14
	500	0.70	3.85	8.12	3.14
Feces	5	9.35	0.26	9.06	1.75
	80	8.43	−0.65	7.39	3.40
	1600	8.69	5.13	9.83	−2.70

The sample of feces was evaluated by high performance liquid chromatography (HPLC) method.

Table S3. Extraction recovery of methyl 3-amino-6-methoxythieno [2,3-b] quinoline-2-carboxylate (PU-48) in biological samples detected by liquid chromatography-tandem mass spectrometry (LC-MS/MS) method (*n* = 3).

Biological Samples	Nominal Concentrations (ng/mL)	Extraction Recovery (%)	RSD (%)
Plasma	0.2	92.72 ± 7.71	8.32
	10	91.25 ± 2.26	2.48
	500	90.15 ± 4.69	5.20
Liver	0.2	87.76 ± 1.46	1.66
	10	107.77 ± 4.85	4.50
	500	96.97 ± 0.99	1.03
Muscle	0.2	95.11 ± 3.31	3.48
	10	100.08 ± 10.08	10.07
	500	88.71 ± 2.75	3.10
Fat	0.2	97.46 ± 5.34	5.48
	10	94.01 ± 2.74	2.92
	500	98.71 ± 0.65	0.65
Urine	0.2	89.93 ± 3.26	3.63
	10	87.66 ± 3.68	4.20
	500	87.45 ± 2.94	3.37
Feces	5	94.42 ± 3.44	3.65
	80	96.15 ± 3.36	3.49
	1600	95.00 ± 6.52	6.86

The sample of feces was evaluated by high performance liquid chromatography (HPLC) method.

Table S4 Ration of peak value between methyl 3-amino-6-methoxythieno [2,3-b] quinolone-2-carboxylate (PU-48) and megestrol acetate (internal standard, IS, 0.2 μg/mL) in blank rat plasma detected by high performance liquid chromatography (HPLC) method (*n* = 5).

**PU-48**	
Actual concentration (μg /mL)	Measured concentration (μg /mL)
0.002	0.0160 ± 0.0021
0.005	0.0307 ± 0.0033
0.01	0.0535 ± 0.0062
0.02	0.1076 ± 0.0051
0.04	0.2077 ± 0.0100
0.08	0.4202 ± 0.0164
0.2	1.0287 ± 0.0655
0.4	1.9283 ± 0.1209
0.8	3.8415 ± 0.1430
1.6	7.6437 ± 0.6361
3.2	15.5144 ± 0.8299

Table S5 Precision and accuracy data of methyl 3-amino-6-methoxythieno [2,3-b] quinolone-2-carboxylate (PU-48) detected by high performance liquid chromatography (HPLC) method in rat plasma (*n* = 5; series per day; 5 days).

Actual Concentration (μg/mL)	Measured Concentration (μg/mL)	Precision (RSD, %)	Accuracy (RE, %)
	Intra-day (*n* = 5)		
0.005	0.0049 ± 0.0003	6.02	−1.95
0.08	0.0785 ± 0.0044	5.57	−1.86
1.6	1.5178 ± 0.0211	1.39	−5.14
	Inter-day (*n* = 25)		
0.005	0.0050 ± 0.0004	7.46	0.13
0.08	0.0830 ± 0.0051	6.17	3.76
1.6	1.5065 ± 0.077	5.11	5.84

RSD: relative standard deviation (%); RE: relative error (%).

Table S6 Stability data of methyl 3-amino-6-methoxythieno [2,3-b] quinolone-2- carboxylate (PU-48) detected by high performance liquid chromatography (HPLC) method in rat plasma (Mean ± SD, *n* = 3).

Stability	Actual Concentration (μg/mL)	Measured Concentration (μg/mL)	Precision (RSD, %)	Accuracy (RE, %)
Light and room temperature for 24 h	0.005	0.0045 ± 0.0003	5.87	−9.278
	0.08	0.0873 ± 0.0011	1.28	9.07
	1.6	1.4299 ± 0.0296	2.07	−10.63
Freeze−thawing three cycles (at −20 °C)	0.005	0.0046 ± 0.0004	9.12	−7.33
	0.08	0.0773 ± 0.0055	7.07	−3.43
	1.6	1.4641 ± 0.0840	5.73	−8.49
Post−preparative stability (at 4 °C for 96 h)	0.005	0.0046 ± 0.0006	12.74	−8.43
	0.08	0.0718 ± 0.0019	2.70	−10.23
	1.6	1.5507 ± 0.0904	5.83	−3.08
Freezing storage stability (at −20 °C for 3 months)	0.005	0.0045 ± 0.0002	4.81	−9.37
	0.08	0.0748 ± 0.0058	7.79	−6.48
	1.6	1.4376 ± 0.0066	4.56	−10.15

Table S7. Plasma concentration - time courses of methyl 3-amino-6-methoxythieno [2,3-b] quinoline-2-carboxylate (PU-48) after single oral administration of PU-48 SNEDDS at the dose of 3, 6 and 12 mg/kg evaluated by the validated LC-MS/MS method in rats (*n* = 6).

Time（h）	Plasma concentration (ng/mL)		
	3 mg/kg	6 mg/kg	12 mg/kg
0.25	11.9 ± 15.5	51.1 ± 51.2	91.1 ± 52.0
0.5	12.6 ± 11.1	52.9 ± 46.8	94.3 ± 49.6
1	11.8 ± 11.2	48.3 ± 32.8	74.8 ± 36.1
2	13.8 ± 16.6	16.8 ± 10.1	19.7 ± 4.2
4	1.1 ± 0.7	2.3 ± 1.2	4.3 ± 3.1
6	0.3 ± 0.2	1.2 ± 0.5	2.1 ± 0.8
8	0.5 ± 0.9	0.6 ± 0.4	1.5 ± 0.5
12	0.1 ± 0.1	0.3 ± 0.2	1.1 ± 0.8
24	ND	0.3 ± 0.3	0.1 ± 0.0
36	ND	0.2 ± 0.1	0.8 ± 0.9
48	ND	ND	0.2 ± 0.0

The lower limit of quantitation (LLOQ) was 0.1 ng/mL detected by the high-performance liquid chromatographic-tandem mass spectrometry (LC-MS/MS) method. ND: not detectable.

Table S8 Mean urine accumulative excretion amount of methyl 3-amino-6-methoxythieno [2,3-b] quinoline-2-carboxylate (PU-48) after oral administration of PU-48 SNEDDS（12mg/kg）in rats (*n* = 6, mean ± SD).

Time Point (h)	PU-48 Concentration (ng /mL)	Excretion Amount (ng)	Accumulative Excretion Amount (ng)	Accumulative Excretion Percentage (%)
0–1	627.9 ± 94.1	61.2 ± 13.4	61.2 ± 13.4	0.0022 ± 0.0005
1–2	376.3 ± 184.2	80.8 ± 41.0	131.8 ± 37.9	0.0047 ± 0.0015
2–4	97.3 ± 20.1	107.7 ± 30.0	203.6 ± 48.4	0.0073 ± 0.0018
4–6	156.3 ± 65.6	141.7 ± 78.0	345.3 ± 107.2	0.0123 ± 0.0038
6–12	83.6 ± 56.6	151.1 ± 94.8	496.4 ± 129.9	0.0178 ± 0.0048
12–24	65.3 ± 94.6	354.3 ± 485.8	850.7 ± 589.4	0.0306 ± 0.0216
24–36	45.0 ± 36.9	207.5 ± 123.7	1058.2 ± 684.1	0.0380 ± 0.0250
36–48	25.6 ± 27.4	158.4 ± 125.3	1216.6 ± 716.3	0.0436 ± 0.0261
48–72	7.5 ± 5.9	103.2 ± 94.5	1319.8 ± 734.0	0.0473 ± 0.0267
72-96	9.0 ± 5.4	138.7 ± 101.2	1458.5 ± 733.1	0.0523 ± 0.0267

Table S9. Mean feces accumulative excretion amount of methyl 3-amino-6- methoxythieno [2,3-b] quinoline-2-carboxylate (PU-48) after oral adminstration of PU-48 SNEDDS （12mg/kg）in rats (*n* = 6, mean ± SD).

Time Point (h)	PU-48 Concentration (μg /mL)	Excretion Amount (μg)	Accumulative Excretion Amount (μg)	Accumulative Excretion Percentage (%)
0–6	0.1	0.1	0.1	0.0022
6–8	0.4	0.4	0.4	0.0128
8–12	6.9 ± 6.5	12.5 ± 14.5	12.5 ± 14.6	0.44 ± 0.51
12–24	9.4 ± 5.3	19.0 ± 11.3	31.5 ± 21.4	1.11 ± 0.73
24–48	1.8 ± 1.9	9.5 ± 9.5	41.0 ± 21.7	1.45 ± 0.74
48–72	0.5 ± 0.7	3.2 ± 4.4	44.3 ± 22.1	1.56 ± 0.75
72–96	0.2 ± 0.2	1.2 ± 1.4	45.5 ± 22.0	1.61 ± 0.74

Table S10. Mean bile accumulative excretion amount of methyl 3-amino-6-methoxythieno [2,3-b] quinoline-2-carboxylate (PU-48) after oral adminstration of PU-48 SNEDDS（12mg/kg）in rats (*n* = 6, mean ± SD).

Time Point (h)	PU-48 Concentration (ng /mL)	Excretion Amount (ng)	Accumulative Excretion Amount (ng)	Accumulative Excretion Percentage (%)
0–1	143.8 ± 72.5	78.5 ± 60.3	78.5 ± 60.3	0.0027 ± 0.0021
1–2	123.1 ± 44.3	75.2 ± 70.3	153.6 ± 115.5	0.0054 ± 0.0041
2–4	84.7 ± 20.1	65.3 ± 39.8	218.9 ± 128.9	0.0076 ± 0.0046
4–6	47.6 ± 19.4	24.5 ± 14.3	234.4 ± 124.7	0.0085 ± 0.0044
6–8	41.1 ± 21.9	18.7 ± 13.4	262.1 ± 11.3	0.0091 ± 0.0042
8–12	33.3 ± 17.4	39.7 ± 45.9	301.8 ± 105.8	0.0105 ± 0.0037
12–24	33.4 ± 15.5	38.1 ± 37.4	339.9 ± 122.8	0.0118 ± 0.0042
24–48	9.4 ± 5.4	10.8 ± 3.2	350.7 ± 124.1	0.0122 ± 0.0043
48–72	5.1 ± 3.7	4.8 ± 4.7	355.5 ± 120.8	0.0123 ± 0.0042
72–96	1.4 ± 0.9	2.0 ± 1.8	357.5 ± 120.1	0.0124 ± 0.0041

© 2018 by the authors. Submitted for possible open access publication under the terms and conditions of the Creative Commons Attribution (CC BY) license (http://creativecommons.org/licenses/by/4.0/).
